# IOATLAS: scanning across the medical horizon

**DOI:** 10.1017/S0266462325100469

**Published:** 2025-08-26

**Authors:** Hannah O’Keefe, Elizabeth Green, Anjum Jahan, Imogen Forsythe, Jane Nesworthy, Sonia Garcia Gonzalez-Moral

**Affiliations:** 1 NIHR Innovation Observatory, Newcastle University, Newcastle-upon-Tyne, UK; 2 Population Health Sciences Institute, Newcastle University, Newcastle-upon-Tyne, UK

**Keywords:** horizon scanning, forecasting, foresight analysis, MedTech, therapeutics, standardization, glossary, software

## Abstract

**Introduction:**

Horizon scanning (HS) is a methodology that aims to capture signals and trends that highlight future opportunities and challenges. The National Institute for Health and Care Research (NIHR) Innovation Observatory routinely scans for medical technologies and therapeutics to inform policy and practice for healthcare in the United Kingdom (UK). To date, there is no standardized terminology for horizon scanning in healthcare. Here, we discuss the development of a data glossary and the IOAtlas web app.

**Methods:**

We extracted data points from 4 years’ worth of NIHR Innovation Observatory HS projects and collated them by technology type and descriptive family. A source repository was established by extracting a list of all sources used in NIHR Innovation Observatory briefing notes between 2017 and 2021. The repository was validated by external HS organizations and experts, and sources were then mapped to the appropriate time horizons. The glossary and repository were converted to an SQLite database format and connected to a free web app, IOAtlas.

**Results:**

After de-duplication and consolidation, a total of 148 data points were included in the glossary. The source repository consists of 149 sources, with 99 percent being compliant with searching for two or more technology types. The final SQLite database contained 35 tables with 36 relationships.

**Conclusions:**

We present a data glossary to provide globalized standardization for the terminology used in HS projects. The glossary can be accessed through the IOAtlas web app. Furthermore, we provide users with an interface to generate downloadable data extraction templates within IOAtlas.

## Introduction

The health and care landscape is continually shifting with the introduction of different types of innovation, including incremental, disruptive, and novel technologies, as well as systems architecture. To keep at the fore, decision makers must seek not only current innovations, but also those that are emerging (1,2). Horizon scanning (HS) is a method within the broader family of foresight methodologies that aims to capture signals and insights that could reveal future changes, challenges, and priorities (1,3). Outputs from HS are used by policy makers, decision makers, regulators, National Health Service (NHS) groups, and commissioning groups, amongst others, to inform the direction and economic implications of healthcare practice (4). HS can be applied to different categories of health and care research. At the National Institute of Health and Care Research (NIHR) Innovation Observatory, we routinely conduct HS in the medical technologies (MedTech) space, covering devices, diagnostic and digital technologies, and the therapeutic space, covering novel and repurposed medicinal agents. Under these categories, we conduct HS covering six types of data: Product Pipeline, Intelligence and Insights, Clinical Landscape, Funding Landscape, Patents scan, and Literature scan. These may be used alone or in combination and often require expert consultation. As a final level, we consider the innovation development pipeline at three different time horizons. The emerging horizon (H3) covers innovations and preclinical applications 5–15 years away from market access. The transitional horizon (H2) can be described as the transition between investigational and nearly established phases, and generally covers clinical trials and early pre-regulatory processes at 0–7 years pre-market access. Finally, the imminent horizon (H1), which is concerned with more mature innovations, covering late-stage pre-regulatory processes, market access approved, and post-market surveillance (5). Post-market surveillance products will often undergo clinical effectiveness and economic evaluation through Health Technology appraisals (HTA). HS for such products provides insights into emerging trends, risks, and opportunities which may drive further innovation (1,6).

The process of HS follows several steps: scoping, protocol and search strategy development, searching, sifting, data extraction, analysis and synthesis, and dissemination. What sources are searched is largely dependent on the technology category, type of HS report, and the time horizon of interest. Similarly, the inclusion and exclusion criteria used at the sifting stage, as well as which data points need to be extracted, will relate to these aspects. Therefore, it is imperative that these are considered at the protocol stage (3).

Standardization and common understanding of the meaning of the data and intelligence presented across different stakeholders form the basis of scientific progress. Customized, ad-hoc selection of relevant data points and their manual data extraction is a time-consuming and resource-intensive task in the HS process. At the NIHR Innovation Observatory, data point selection is conducted by a single member of the team, piloted by the whole team, and iteratively adapted as the project progresses. It takes approximately 2 weeks to construct a data extraction template and pilot it in the first instance. However, templates are rarely constructed from scratch. A naïve HS center with little experience would find this process far more time-consuming. Furthermore, a lack of standardization may lead to unjustified heterogeneity in outputs and a barrier for quality appraisal and assurance processes. There is a tendency for organizations to use their own classification and terminology, which can result in miscommunication. For example, the word “subgroup” can be taken in multiple ways, such as a patient cohort based on a population characteristic or a characteristic of the condition, or a cohort of devices based on technical characteristics. Pre-determined data points, with clear definitions, mapped to different types of technologies and research questions, will provide a framework for enhanced HS outputs.

A dictionary is a reference work that lists words, usually in alphabetical order, and provides their meanings and pronunciation. Dictionaries have existed since ancient times and are the basis for communication and disambiguation of terminology. Different scientific disciplines have used the same approach to create glossaries, which are a standardized and commonly agreed set of terms pertaining to a particular discipline that are usually presented in alphabetical order and appended to publications as an aid for their interpretation. In the field of HS for innovation in health and care, a standardized glossary of terms is lacking. A data glossary will support the interpretation of intelligence by a range of different stakeholders, and it will provide an opportunity to explore automation of data collection.

## Aims and objectives

The aim of this project was to retrospectively identify the main data points used to inform decision-making for a comprehensive range of healthcare innovative technologies. Based on this aim, the objectives were to:

1. Associate these data points to specific healthcare innovative technologies (e.g., diagnostics)
2. Map data points to the main sources of information
3. Provide definitions of each data point
4. Create a platform to consult and access these data points (i.e., online database, report on IO website).

## Methods

### Elaboration of the data glossary

Data points were initially identified from previous HS data extraction forms, which included eight medicine HS projects and 16 MedTech HS projects completed between 2020 and 2023. Data points from the two strands were first extracted separately, with all data from each scan being assigned a unique color code. Extracted data points from HS were de-duplicated and consolidated, then grouped into 11 unique families.

#### Data merging

The process of de-duplication and consolidation produced a clean list of data points. At this point, the color coding representing each HS topic was removed, and the data points extracted from the medicines scans and MedTech scans were merged into one workbook. The process of de-duplication and consolidation was repeated to produce a final set of data points. The final data points were then grouped into new families (Table 1).

**Table 1.** Family names are used to categorise merged data points

Family name
Industry
Product/intervention
Diagnostic performance
Indication
Product implementation
General information
Strength of evidence
Clinical trial
Clinical pathway
Regulatory information
Quality assurance

#### Defining data points

To define the data points, trusted sources such as Citeline and ClinicalTrials.gov were utilized to establish consistent definitions. These sources allowed for the identification of highly scan-specific data points, which could be incorporated on an ad hoc basis as needed. This process further enabled the classification of data points into primary and secondary categories, ensuring a structured and comprehensive dataset.

### Compilation of the repository of sources

HS relies on robust search methodology for the identification of signs of innovation. For the NIHR Innovation Observatory, these signs may be interpreted as new and innovative healthcare technologies in development or market-ready. For the efficient and timely identification of these technologies is crucial that we use the most appropriate and up-to-date sources of information.

The compilation of these sources of information has been undertaken alongside the development of internal tools, systems, outputs, and databases, and is constantly being updated. The first stage of compilation started in April 2017 when the NIHR Innovation Observatory was launched. A list of general sources of information for the practice of healthcare innovation HS was extracted from the most up-to-date version of the EuroScan Methods Toolkit at that time (7). Thirty-two listed sources were appraised for currency, relevancy, and accessibility, and grouped according to the following classification:

Depending on the accessibility and level of information and data processing, these sources were classified as:

1. Primary, those that are freely available and come directly from the originator, that is, company news. Primary sources included interviews, engagement with experts, manufacturers, professional colleges, professional and patient groups, websites, industry publications, specialist journals, newsletters, clinical trials, patent registries, hospital data, social media sources, and financial reports.
2. Secondary, sources that provide information originated by another entity, that is, journals or databases. Secondary type of sources included bibliographic databases (Medline, PubMed, ISI Web of Science), regulatory authorities, Google and Google Scholar, specialist databases, Reuters, Medscape, research funding databases, commercial databases, internal databases (defined as those created for internal use of the organization), and conference abstracts/reports.
3. Tertiary, those that generate new information from analysis of primary sources. The group of tertiary sources comprised HS reports and HTA reports.

A new repository was created containing sources that were still current and freely accessible at the time of assessment.

Between 2017 and 2021, the NIHR Innovation Observatory produced a total of 693 technology briefings that entered the technology appraisal programs at the National Institute for Health and Care Excellence (NICE) in the United Kingdom (UK) (6). In 2021, analysts manually extracted the sources from all these briefings and produced a single de-duplicated list of sources most commonly used and consulted for the preparation of those technology briefings. The results from this analysis identified 99 unique sources that were added to the ongoing repository of sources. This constituted the second stage of the process of building a source repository.

The third stage consisted of the validation of this repository by external HS organizations and experts. The International HS Initiative (IHSI) is an alliance of international HS organizations that share common goals and vision (8). The NIHR Innovation Observatory took an advisory role in the IHSI Medical Devices working group, which aimed to develop an HS solution for their medical health technologies workstream. As part of Objective 6 of their working group plan, the NIHR Innovation Observatory shared the list of compiled sources with the rest of the working group members and asked for feedback, validation, and/or addition of sources used by other organizations in their HS practice. The final sources were further classified by type of source, type of technology most likely to provide information about, and mapped against pre-established time horizons agreed amongst the members of the working group.

### Mapping of sources to horizons

To streamline the selection of sources, we aimed to indicate the likelihood of a particular source to retrieve the type of signals needed to satisfy a given stakeholder need for a technology scan. We used “time horizon” as an estimate of how early the technology might be from market access (MA), assuming a linear product development pathway. The time horizon endpoint is the moment the product gains MA in any country or regulatory jurisdiction.

### Possible values

- **Innovation:** 20 to 10 years ahead of product MA: classed as a very early technology stage, this time horizon will usually apply to signals emerging from patents or very early scientific discoveries.
- **Preclinical to early clinical** (10 to 5 years ahead of product MA) classed as early stage, this time horizon will usually apply to signals emerging from pre-clinical studies or early clinical trials (phase I), news of new product development, or company press releases about new technologies in their pipeline.
- **Clinical trials** (5 to 2 years ahead of product MA): classed as the technology assessment stage, this is usually the timeframe used to notify HTA bodies ahead of product MA.
- **Initiation of regulatory processes and Market Access Application (MAA)** (3 to 1 years ahead of product MA approval), these would be technologies at the late stage or very late stage of development.
- **Post-market surveillance**: all evidence synthesis sources where systematic literature reviews or health technology appraisals are the main source. These sources are often searched to complement the strength of signals identified by other means, not necessarily to identify emerging technologies.

We considered these possible values under the emerging horizon (innovation and preclinical stages), transitional horizon (clinical trials and start of regulatory processes), and imminent horizon (MAA and Post-market surveillance). Columns were added to the repository to mark the type of scan and which of the three horizons the sources applied to. Each source was evaluated for these metrics by a single reviewer (HOK). The data for type of scan, category of scan, and time horizon were separated into individual tables, unpivoted, and flattened in Microsoft (MS) Excel to create tables which could be imported into MS Access.

### Online database proof of concept model

Python Streamlit was used (9), in conjunction with Pandas and Numpy packages (10,11), to create a searchable web interface for the data glossary. Initially, a basic unpivoted MS Excel table with Family, Data Point, and Details columns was used as proof of concept. Briefly, a Streamlit app was created, which had a hierarchical structure of family heading, data point subheadings, and a drop-down description for each data point. A drop-down menu was provided to search by family, and a text input box for searching by data point.

### Data schema

MS Access was used to build a relational database that connected the data glossary and the source repository (12). This allowed for fine-tuned filtering of data, which could be used to build tailored outputs.

Individual tables were created for each data point family in the glossary and the sources in the repository. Alongside these were tables for the type of HS report, category of HS, and time horizon. Each table was connected by ID numbers to allow for one-to-many relationships. For example, a single source may be applicable to three types of HS, forming a 1:3 relationship.

To make the database more compact and easier to embed in the Streamlit app, it was migrated to SQLite using RebaseData, an online relational database conversion platform (13).

### Public glossary and template generator

The proof-of-concept model was reconfigured to enable data to be called from the SQLite database using the sqlite3 package (14). Instead of calling the data from MS Excel into a global variable, the data is fed directly from the SQLite database into variables within the functions. This means that functions are only able to iterate over the necessary data rather than requiring the whole dataset, reducing the computing power required and speeding up the process.

A Template Generator page was established, which utilized the pandas and sqlite3 packages to query the data and relationships. The “INNER JOIN” function was used to confirm relationships between tables, and the “WHERE” clause was used to query across relationships.

Drop-down menus were provided to enable filtering by type of scan report, category of scan, and time horizon. After the user makes selections, a button click generates a list of related data points, subject-specific data points, and sources, displayed as three separate tables on the screen. There is a download button available to generate an MS Excel workbook containing a worksheet with transposed version of data points table (Rows: Data points, Descriptions), a second worksheet with the sources table (Columns: Source Name, Source Type, URL, Access, Source Tag, Location Tag), and a third worksheet with subject specific data points (Rows: Data points, Descriptions).

## Results

### Data glossary

**Figure 1.**
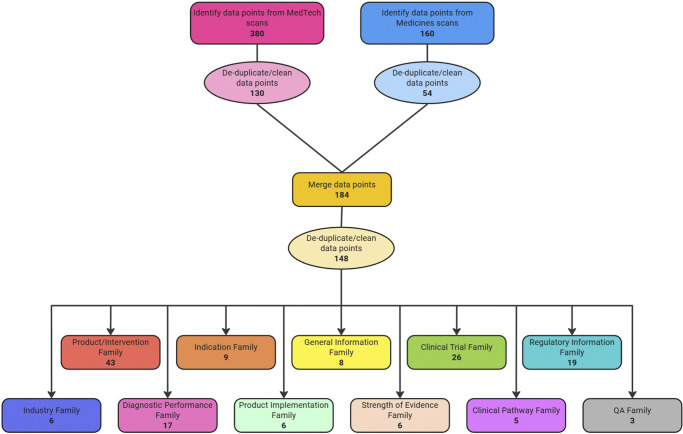
A flow chart depicting the number of data points at each stage of the process.

A total of 380 data points were returned from MedTech scans and 160 from medicines scans. De-duplication left 130 data points from MedTech scans and 54 from medicines scans. The merging of MedTech and medicine scans collected a total of 184 data points. A final total of 148 data points remained following de-duplication of the merged set (Figure 1). The largest families of data points were the product and information family (*n* = 43), clinical trial family (*n* = 26), and regulatory information family (*n* = 19). The families with the fewest amount of data points were the clinical pathway family (*n* = 5) and QA family (*n* = 3).

### Source repository

**Figure 2.**
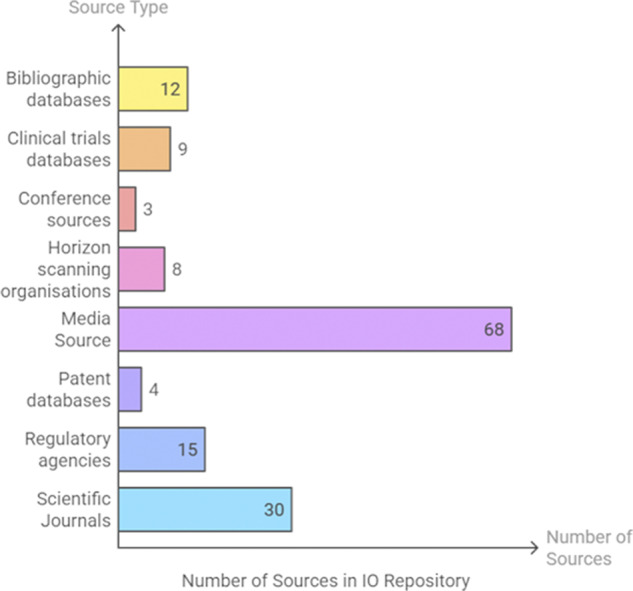
Distribution of sources in the IO repository by type.

The final list consisted of a total of 149 different sources. The top three sources of signal detection were media sources (68/149), such as newspapers, blogs, specialist publications, press releases, or research news; scientific journals (30/149), and regulatory agencies (15/149). Figure 2 presents the full distribution of sources by type.

Our mapping against the type of technology showed that the majority of the sources (83/149) provided access to information on all types of technologies, including pharmaceuticals, medical devices, diagnostics, and digital; 65 sources were dedicated exclusively to medical devices, diagnostics, and digital interventions while only one source was dedicated to medical devices alone.

### Access/SQLite schema

**Figure 3.**
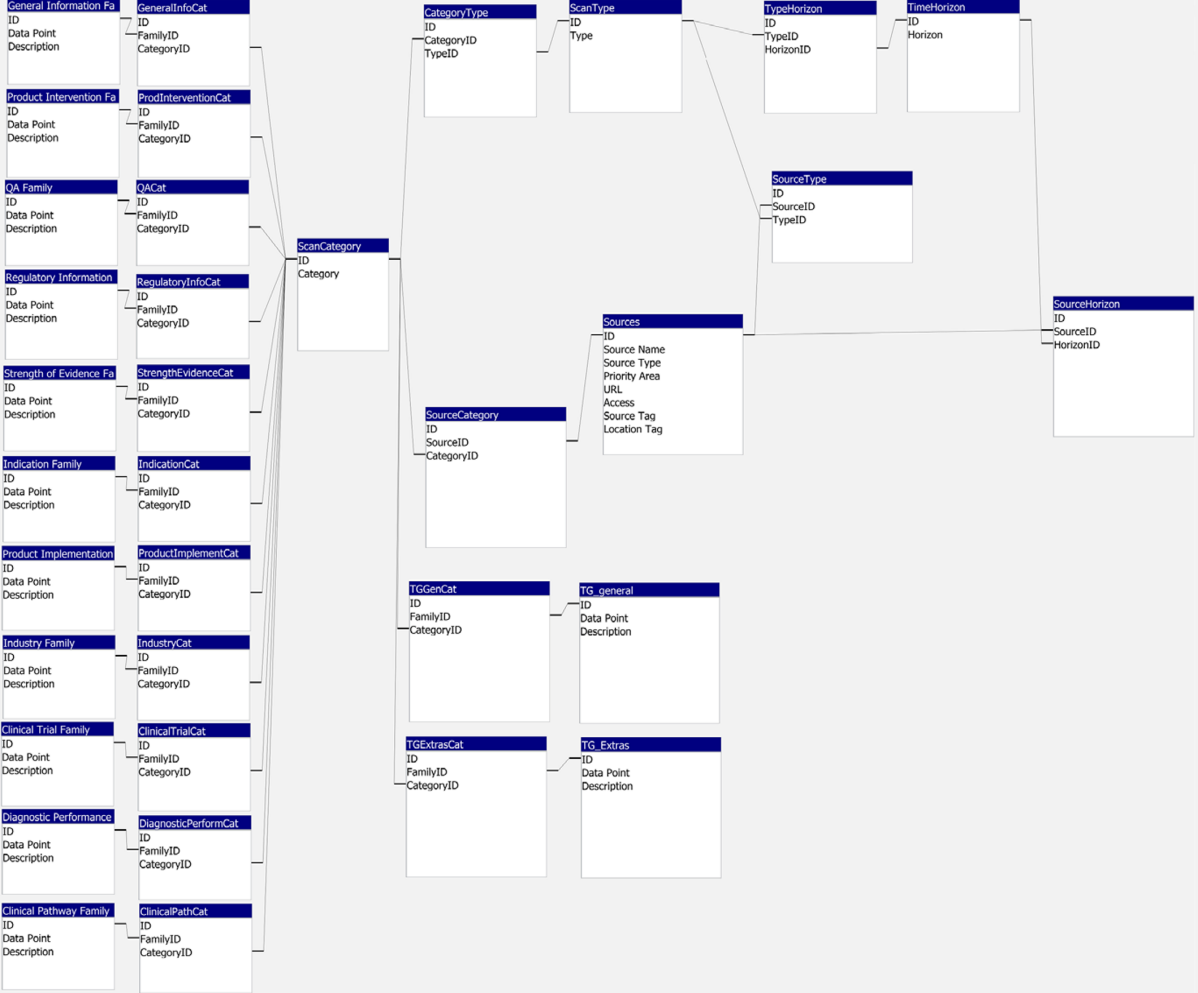
Database schema designed in MS Access, including 35 tables and 36 relationships.

The MS Access database consisted of 35 tables and 36 relationships (Figure 3). A table was created for each of the data point families, type of scans, time horizons, sources, and category of scan (*n* = 17 tables). These were supplemented with connector tables (*n* = 18) to form the relationships.

Conversion from MS Access to SQLite maintained these relationships and resulted in a reduction in file size from 3724 to 228 KB. The SQLite file was embedded in the Streamlit app folder, mitigating the need for independent database hosting.

### App design

**Figure 4.**
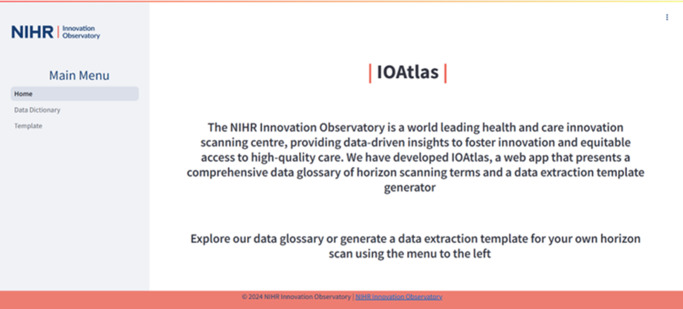
App design using Python, Streamlit, and CSS, with three “click and go” pages: a Home page, a Data Glossary page, and a Template Generator page. Available at: https://ioatlas.nihrio.com/

The app design utilized basic concepts in Python and Streamlit, with CSS and markdown for styling. We designed three “click and go” pages: a Home page, a Data Glossary page, and a Template Generator page. The use of three pages meant we were able to make the webapp intuitive to use, introduce the webapp, give explanations where necessary, and remove the user’s need to interact with complex backend coding. Styling ensured consistency with NIHR Innovation Observatory branding, making the webapp a recognizable product (Figure 4). It also allowed us to develop a styling template (.CSS file) for future Streamlit web apps.

## Discussion

We created IOAtlas, a free-to-access online tool to help researchers build capacity within HS projects by delivering a standardized glossary of terminology for data and intelligence (Available at: https://ioatlas.nihrio.com/). We aimed to reduce time-consuming and resource-intensive ad-hoc selection of relevant data points and remove heterogeneity in HS processes for health technologies (15). The NIHR Innovation Observatory has a wide range of stakeholders who will benefit from this standardization and shared understanding. IOAtlas is a testbed and has far-reaching implications, as a lack of standardization has until now remained a challenge for the system and various players within the system. Addressing the lack of standardization and presenting it in a way that enables those from multiple locations and backgrounds to access the resource without limitations was at the heart of this project.

IOAtlas is a twofold web app. Firstly, we have supplied a comprehensive glossary, describing each of the data points that are sought by the NIHR Innovation Observatory during data extraction of HS projects (5). This glossary was designed to reduce ambiguity in terminology and provide openness and transparency to our processes. It acts as a reference point for stakeholders and interested parties, as well as acting as a guide for those wishing to perform their own HS. Secondly, we expanded the application of the glossary to develop the template generator, providing a robust and reproducible set of data extraction points and potential search sources that are aligned with the type of HS and time horizon of interest. This minimizes the need for manual generation, which may prove cost-effective by reducing time-on-task and redistributing resource allocation, which has been previously identified as a barrier to implementing HS (15). This is particularly important in resource-limited settings. IOAtlas is freely available, allowing access to information that may otherwise be inaccessible behind paywalls and could lead to a reduction in staffing outlays. There is a growing international interest in HS, with many institutions beginning to implement their own HS systems. Those who are new to HS processes may benefit from having access to resources from experienced HS centers. The IOAtlas glossary provides shared terminology that could limit misinterpretation when translating between languages.

The presentation and acceptance of a universal glossary underpins the opportunity to explore automated data extraction. Standardization of terms allows the interpretation and mapping of synonyms and antonyms whilst reducing ambiguity. It will also foster cross-platform standardization, permitting greater collaboration and automation of processes (16). Standardized reporting of HS methodologies is needed in the field, particularly when using automation techniques underpinned by artificial intelligence (AI) (1). Equally, evaluation is an integral part of the development lifecycle to make IOAtlas universally applicable and meaningful, with or without automation. Comprehensive, effective evaluation of this tool will require a longitudinal feedback period, whereby NIHR Innovation Observatory analysts and stakeholders can assess the application across different types of HS projects covering a broad range of topics. Although initial evaluation will be based on NIHR Innovation Observatory usage and stakeholder engagement, further evaluation from research groups, both experienced and new to HS, will help us improve the content and accessibility of IOAtlas.

As part of a long-term strategy, an exhaustive evaluation plan will be designed to ensure we capture recommended improvements and impact markers to assess the effectiveness of using IOAtlas to support HS processes. We are designing a process for collating and approving new terms to be added to the glossary based on user feedback. This is likely to take the form of a suggestion box embedded in the web app, with an annual review panel discussion. Furthermore, the NIHR Innovation Observatory is in the process of exploring how large language model capabilities map to the types of outputs we produce and the challenges we face in creating them. This includes considering how large language models or other text mining techniques may be used to populate data extraction sheets generated by IOAtlas, utilizing data from our core data engine OpenScan. IOAtlas is a testbed to help define shared terminology and align therapeutic and MedTech schemas across OpenScan and the broader national data infrastructure being developed by the NIHR Innovation Observatory. As part of this work, we are developing a list of reporting items for AI use in HS. This will be embedded into IOAtlas and downloaded alongside the data extraction template to ensure a robust and transparent report is available to all users.

Limitations of IOAtlas include the need to continuously monitor, validate, and add new terminology to the glossary and new sources to the source repository. It also entails removal of obsolete terminology and sources, and maintenance of information regarding current sources, such as up-to-date URLs and what content the sources cover. This is a time-consuming endeavor and requires dedicated resources. As mentioned above, adding a suggestion box to IOAtlas may be a way of overcoming this limitation. A suggestion box would allow a collaborative approach to the continuous development of this tool, whereby users drive the content and functionality through shared ideas and collective intelligence. It would also allow for maintenance and removal, as users would be able to flag issues such as broken web links. At this time, we are initiating a 6-monthly review of IOAtlas to ensure the web app is up-to-date and maintained. This is subject to change with the finalized evaluation plan.

## Conclusions

HS is a core activity in health and social care planning. IOAtlas has been designed to give structure and standardization to the terminology used in this context and across the NIHR Innovation Observatory’s national data infrastructure. The robust methodologies for producing data-driven insights used by the NIHR Innovation Observatory have underpinned the development of IOAtlas. This framework for enhanced HS provides a reference point that will aid interpretation of intelligence and minimize unjustified heterogeneity in outputs by ensuring accessibility and transparency.
